# Analysis of Retinol Binding Protein 4 and *ABCA4* Gene Variation in Non-Neovascular Age-Related Macular Degeneration

**DOI:** 10.3390/diagnostics13142411

**Published:** 2023-07-19

**Authors:** Hung-Da Chou, Yih-Shiou Hwang, Kuan-Jen Chen, Wei-Chi Wu, Laura Liu, Shyh-Tyan Ou, Webber Liao, Cheng-Chi Wang, Tom Lin, Chi-Chun Lai

**Affiliations:** 1 Department of Ophthalmology, Chang Gung Memorial Hospital, Linkou Main Branch, Taoyuan 33305, Taiwan; 2 College of Medicine, Chang Gung University, Taoyuan 33305, Taiwan; 3 Department of Statistics, National Taipei University, Taipei 22102, Taiwan; 4 Lin BioScience Inc., Taipei 11071, Taiwan; 5 Department of Ophthalmology, Chang Gung Memorial Hospital, Keelung 204011, Taiwan

**Keywords:** visual cycle, retinol, lipofuscin, retinal pigment epithelium, macular degeneration

## Abstract

Age-related macular degeneration (AMD) may be associated with *ABCA4* variants and is characterized by the accumulation of visual cycle-byproduct lipofuscin. Reducing retinol-binding protein 4 (RBP4), a retinol transporter protein, may reduce lipofuscin production. This study aims to assess the associations between plasma RBP4, the *ABCA4* variation, and AMD severity. Sixty-seven participants were grouped into healthy/mild AMD (*n* = 32) and severe AMD (*n* = 35) groups. The latter group was older than the former group and had higher levels of RBP4 (36.8 ± 8.3 vs. 30.4 ± 7.0 μg/mL, *p* = 0.0012). The ten participants with six *ABCA4* linked-variants had higher RBP4 than those without (37.8 ± 7.7 vs. 32.4 ± 7.9 μg/mL; *p* = 0.026), and eight of them had severe AMD. Univariate analyses showed that severe AMD was related to older age (OR, 1.26; 95% CI, 1.13–1.40; *p* < 0.0001) and to higher RBP4 levels (OR, 1.12; 95% CI, 1.04–1.20; *p* = 0.003), whereas the linked *ABCA4* variants had no associations. After adjustment, however, only age remained significantly associated with severe AMD. This pilot study shows a trend of higher plasma RBP4 levels in severe AMD or the *ABCA4*-linked variants, and further age-matched studies are warranted.

## 1. Introduction

Age-related macular degeneration (AMD) is an irreversible degenerative eye condition that results in retinal geographic atrophy and eventual blindness. AMD is characterized by the accumulation of toxic lipofuscin near retinal pigment epithelial (RPE) cells, which may lead to the atrophy of RPE cells and photoreceptors [1]. Lipofuscin is composed of materials including *N*-retinylidene-*N*-retinyl-ethanolamine (A2E), which is an all-*trans* retinol derivative produced as a byproduct of the visual cycle. Controlling all-*trans* retinol concentration and A2E production has been used as therapeutic targeting for managing AMD [2,3].

Retinol binding protein 4 (RBP4) is a transporter protein that recruits all-*trans* retinol to the retina. RBP4 is associated with aging [4], metabolic syndromes [5], and cardiovascular diseases [6]. It has also been suggested that RBP4 may be directly involved in the pathogenesis of retinal diseases [7,8]. A reduction in RBP4 levels may reduce the input of all-*trans* retinol to the visual cycle, which in turn reduces the formation of A2E and lipofuscin [7]. In a study of diabetic retinopathy, RBP4 levels in patients with diabetic retinopathy were 1.6-fold higher than those in normal participants [8]. In addition, preclinical and clinical studies have demonstrated that blocking RBP4 slows the progression of retinal disorders [9]. Based on these findings, RBP4 may be a potential therapeutic target and diagnostic biomarker for retinal degenerative diseases, including AMD.

ABCA4 is an ATP-binding cassette (ABC) transporter localized on the membrane of the outer segment discs of photoreceptors. ABCA4 transports all-*trans* retinal across the disc membranes for conversion to a less reactive form of all-*trans* retinol. Dysfunctional ABAC4 is the primary factor in the pathogenesis of Stargardt disease, a juvenile form of macular degeneration that is also characterized by atrophic lesions triggered by the accumulation of lipofuscin. Studies have also identified alterations in the *ABCA4* gene in AMD patients in addition to patients with Stargardt disease [10]. Certain *ABCA4* variants may enhance susceptibility to AMD, while other variations may be associated with a younger age of AMD presentation [11].

However, there has been limited research on the role of RBP4 in AMD. The purpose of the present pilot study is to explore the relationships among plasma RBP4, *ABCA4* variants, and AMD.

## 2. Materials and Methods

Healthy and AMD participants were recruited for this study at the Ophthalmology Department, Chang Gung Memorial Hospital, Linkou, Taiwan. For the AMD participants, the inclusion criteria were an age greater than 50 years and a diagnosis of AMD in the primary eye as defined by the Age-Related Eye Disease Study (AREDS) Research Group [12]. For the healthy participants, participants aged over 50 years of age with no eye disease and a best corrected visual acuity of 20/20 were included. Participants with active hepatitis or chronic kidney disease and pregnant females were excluded from the study. Demographic data, such as age, height, weight, and body mass index (BMI), and the presence of systemic conditions, including obesity (waist/hip ratio > 0.90 for males or 0.85 for females or BMI > 30 kg/m^2^), hyperlipidemia (triglyceride level > 150 mg/dL or high-density lipoprotein cholesterol < 35 mg/dL for males or <39 mg/dL for females), metabolic syndrome [13], prediabetes [14], diabetes mellitus, hypertension (blood pressure ≥ 140/90 mmHg), cardiovascular diseases [15], and non-alcoholic fatty liver diseases and steatohepatitis, were collected from medical records. All participants signed an informed consent form.

### 2.1. AMD Severity Grading

Data on age at AMD diagnosis were collected from medical records, and AMD disease duration was calculated as the time between diagnosis and study enrollment. Color fundus photography (nonmyd 8s; Kowa Company, Ltd., Nagoya, Japan), fundus autofluorescence (Spectralis HRA2; Heidelberg Engineering, Heidelberg, Germany), and spectral-domain optical coherence tomography (Spectralis HRA2; Heidelberg Engineering) were used to grade AMD severity according to the definitions of the AREDS classification [11]. Images were classified by experienced ophthalmologists (K.-J.C., W.-C.W., Y.-S.H., and H.-D.C.) and verified by a senior ophthalmologist (C.-C.L.) at the Chang Gung Memorial Hospital, Linkou. Briefly, AREDS category 1 was defined as no druse or a few (5–15) small (<63 µm) drusen and no pigment changes. AREDS category 2 was characterized by pigmentary changes and/or several small or a few intermediate-sized (63–124 µm) drusen or no pigmentary changes but with several small or a few intermediate-sized (63–124 µm) drusen. AREDS category 3 was characterized by extensive (20 soft or 65 hard without any soft) intermediate-size drusen and/or ≥one large (>125 µm) druse and/or geographic atrophy not involving the macula. AREDS category 4 involved the advanced dry form with geographic atrophy involving the macula or the exudative form with choroidal neovascularization in one eye. For the purpose of analysis, healthy individuals and participants with AMD category 1 or 2 were grouped into the healthy/mild AMD group, and participants with AMD category 3 or 4 were grouped into the severe AMD group.

### 2.2. Plasma RBP4 Level Measurement

Peripheral venous blood was drawn from the study participants. The samples were separated into equal aliquots of buffy coat and plasma for storage in −80 °C freezers until analysis. The plasma apo-RBP4 concentration was measured using a commercial enzyme-linked immunosorbent assay kit (R&D System, Minneapolis, MN, USA). The assay was validated with commercial human RBP4 recombinant protein (R&D System) and healthy human plasma samples at multiple concentration points, including the assay’s maximum and minimum detection levels. The detection range of this assay was 0.224–100 ng/mL, and the intra-assay and inter-assay coefficients of variation for RBP4 were 5.25% and 4.54%, respectively.

### 2.3. ABCA4 Variant Analysis

DNA was isolated from buffy coat samples using a QIAamp DNA mini kit (QIAGEN, Venlo, The Netherlands). Next-generation sequencing of the DNA samples was performed on a MiniSeq with an AmpliSeq custom panel designed by Illumina (Illumina, San Diego, CA, USA). Sequence data were analyzed, and variant calling was performed using BaseSpace (Illumina). Sequence alignment was performed using BWA (0.7.13) on the reference genome GRCh37/hg19 and analyzed using samtools (1.3) and Picard (2.1.1) [16,17,18]. Variant calling was performed using gatk (1.6) [19]. The population databases dbSNP and ClinVar were consulted for curated information based on existing studies, and allele frequencies were obtained from the gnomAD and TOPMed databases [20,21,22,23]. Quantitative trait association modeling was performed using PLINK (1.90) [24].

### 2.4. Statistical Analyses

Data are presented as the means ± SD. Differences in categorical variables among the groups were analyzed using the χ^2^ test or Fisher’s exact test. Continuous variables were compared using the *t* test or the Mann–Whitney test, and simple linear regression analysis was conducted. To identify the factors associated with severe AMD, univariate and multivariate models were constructed with the factors that might affect the condition of AMD. The details of *ABCA4* genetic analysis are described in the above section of *ABCA4* variant analysis. All statistical analyses were performed with R (3.6.0) [25] and RStudio (1.1.442; RStudio Inc., USA) and confirmed with SAS software (Version 9.4, SAS Institute Inc., USA). A *p* value less than 0.05 was considered significant.

## 3. Results

### 3.1. Cohort Characteristics

A total of 67 eligible participants (32 and 35 participants in the healthy/mild AMD and severe AMD groups, respectively) were recruited between March 2018 and May 2019. Table 1 shows the demographics and clinical features of the study cohort. The average age of 72.6 ± 7.7 years for the severe AMD group was significantly older than the age of 58.8 ± 7.5 years for the healthy/mild AMD group (*p* < 0.0001). The average age of AMD diagnosis was also older in the severe AMD group (69.7 ± 7.5 years) than in the healthy/mild AMD group (64.6 ± 7.7 years), although the difference was not statistically significant (*p* = 0.06).

There was no significant difference in the gender makeup (*p* = 0.80), and the BMI was similar between the two groups (*p* = 0.53). The proportion of obese participants was also similar in both groups (*p* = 0.48). None of the participants had cardiovascular diseases, whereas in the severe AMD group, there was a higher proportion of participants with metabolic syndrome (51%) than in the healthy/mild AMD group (28%, *p* = 0.08).

### 3.2. Association between Plasma RBP4 Levels and AMD Severity

**Figure 1:**
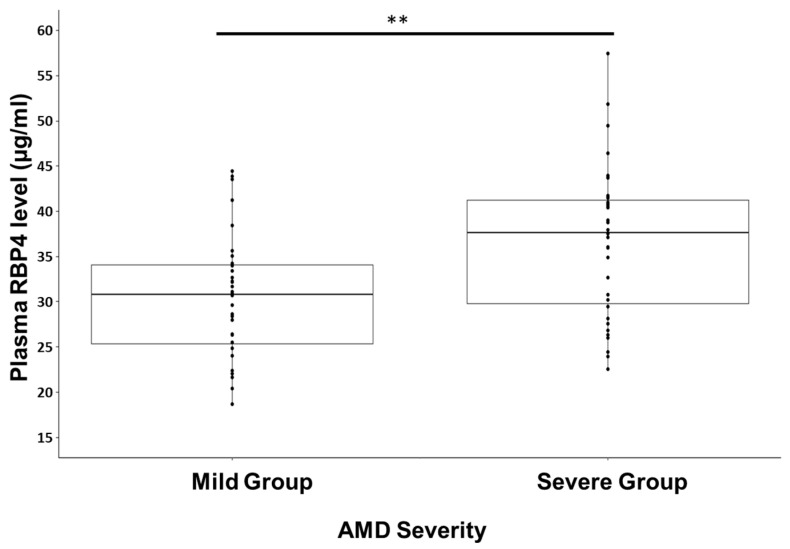
Plasma retinol binding protein 4 (RBP4) levels in the healthy/mild age-related macular degeneration (AMD) and severe AMD groups. ** *p* < 0.01.

The severe AMD group had a significantly higher RBP4 level than the healthy/mild AMD group (36.78 ± 8.3 and 30.43 ± 7.0 µg/mL, respectively; *p* < 0.0001) (Figure 1). Linear correlation analysis showed that the RBP4 levels were significantly and positively associated with age (*p* < 0.0001), AMD severity (*p* < 0.01), and BMI (*p* < 0.05). RBP4 levels were not associated with age at AMD diagnosis.

### 3.3. Associations of ABCA4 Variants with AMD Severity and RBP4 Levels

The *ABCA4* gene of AMD participants was sequenced to identify variants in this population. Thirty-one variants were identified in AMD participants, including five missense mutations, one intronic splice acceptor variant, and one 3-prime UTR variant (Table 2). Twenty-five variants were heterozygous in more than 90% of the carriers, and only two variants were homozygous in the majority of the carriers. Eighteen of the identified variants were consistent with those identified by previous publications [11,26,27,28,29,30], whereas thirteen variants were novel.

Compared to allele frequencies reported in the gnomAD and TOPMed databases, 24 variants appeared at a higher allele frequency in the current cohort. For example, the missense mutation rs1800549 was found in 8 of the 47 AMD patients (17%), which is a higher frequency than the global allele frequency of 0.34%. In addition, many of these variants were found in participants with AREDS category 3 or 4 instead of category 1 or 2 AMD (33–100%). These results imply that the participants with AMD, especially severe AMD, might possess more *ABCA4* variants.

Furthermore, 7 variants were associated with significantly higher RBP4 levels. The 16 participants with dbSNP ID: rs1801555 and the 10 participants with 6 linked variants (namely, dbSNP IDs: rs4147863, rs2275029, rs1800739, rs4147857, rs4147856, and rs1801574) had significantly higher RBP4 than participants without these variants. In addition, 13 of the 16 (81%) participants with rs1801555 and 8 of the 10 (80%) participants with the linked variants had severe AMD. Two participants who possessed the six linked variants exhibited high RBP4 levels (34.3 and 43.5 µg/mL) but had only AREDS category 2 AMD. When investigated further, both participants were newly diagnosed AMD patients upon study recruitment; therefore, we speculated that they may have a high likelihood of developing more severe AMD in the future and suggested close follow-up.

### 3.4. Exploring the Factors Associated with Severe AMD

The factors that potentially have a relationship with severe AMD (AREDS categories 3 and 4) were analyzed in univariate and multivariate logistic regression models (Table 3). In univariate analysis, age was positively and significantly associated with severe AMD (OR, 1.26; 95% CI, 1.13–1.40; *p* < 0.0001). Plasma RBP4 levels were also positively and significantly associated with severe AMD (odds ratio [OR], 1.12; 95% confidence interval [CI], 1.04–1.20; *p* = 0.003). Female sex, BMI, and metabolic syndrome had no association with severe AMD. The six linked *ABCA4* variants that showed significantly higher plasma RBP4 levels also showed no association with severe AMD status.

Furthermore, multivariate models were constructed using age, sex, BMI, metabolic syndrome, and plasma RBP4 level. In the first multivariate model, age remained the only factor significantly associated with severe AMD (OR = 1.27; 95% CI, 1.13–1.44; *p* < 0.0001), whereas plasma RBP4 level (µg/mL) was no longer associated with severe AMD (OR = 1.06; 95% CI, 0.96–1.17; *p* = 0.26).

We further explored using plasma RBP4 levels between 32 and 36 µg/mL as a cutoff value for the association analysis of severe AMD. This was based on the mean RBP4 level of the whole study group (33.75 µg/mL) and the severe AMD group (36.78 µg/mL). The univariate analysis showed that a plasma RBP4 level of 36 µg/mL (OR, 8.13; 95% CI, 2.51–26.32; *p* = 0.0005) was better at differentiating between the healthy/mild AMD and severe AMD groups than 32 µg/mL (OR, 3.18; 95% CI, 1.17–8.70; *p* = 0.024), 33 µg/mL (OR, 4.22; 95% CI, 1.52–11.76; *p* = 0.006), 34 µg/mL (OR, 5.75; 95% CI, 1.99–16.67; *p* = 0.0012), or 35 µg/mL (OR, 6.06; 95% CI, 2.04–17.86; *p* = 0.0011).

Another multivariate model was constructed based on the cutoff value of RBP4 at 36 µg/mL. Similar to the abovementioned model, this model showed that after adjustment for age, sex, metabolic syndrome, and plasma RBP4 level, the only factor that was significantly associated with severe AMD was age (OR, 1.27; 95% CI, 1.12–1.43; *p* < 0.001).

## 4. Discussion

AMD is one of the most prevalent eye diseases in the elderly population and is characterized by the presence of lipofuscin in the RPE layer [1]. After photoexcitation, all-*trans* retinaldehyde attached to rhodopsin is released and forms *N*-retinylidene phosphatidylethanolamine (A2PE). A2PE is normally processed and reduced to all-*trans* retinol for reuse in another phototransduction reaction in healthy individuals. However, A2PE gradually accumulates with age, allowing A2PE to form A2E, and the latter is a component of lipofuscin and induces cytotoxicity in photoreceptor and RPE cells [2]. A potential treatment for preventing the toxicity of A2E is reducing the formation and accumulation of A2E by modulating the visual cycle [3]. One approach is to directly inhibit the expression of visual cycle-related proteins [32]. Alternatively, A2E formation can be modulated by reducing the all-*trans* retinol supply to the retina; this can be achieved by inhibiting RBP4, which is the primary transporter protein of all-*trans* retinol.

RBP4 is a 21-kDa protein with a single binding site for all-*trans* retinol [33]. It is primarily produced in the liver and delivers all-*trans* retinol to peripheral tissues. Most research on RBP4 to date is in the fields of metabolic syndromes and cardiovascular diseases [5,6]. A study on acute ischemic stroke found a significantly higher serum RBP4 level in stroke patients than in normal controls (28.9 versus 23.7 μg/mL) [34]. Another 10-year study of 352 children showed significantly higher baseline RBP4 levels in children with persistent metabolic syndrome (42.1 µg/mL) than in those who never exhibited any sign of metabolic syndrome (32.7 µg/mL) [5]. Furthermore, baseline RBP4 levels were able to predict hyperglycemia, elevated triglyceride levels, elevated blood pressure, and insulin resistance in the study [5]. Our study cohort also showed a similar trend consistent with these findings, with a higher proportion of metabolic syndrome (51%) and higher plasma RBP4 levels (36.8 µg/mL) found in the severe AMD group than in the healthy/mild AMD group, which had a lower metabolic syndrome prevalence of 28% and plasma RBP4 level of 30.4 µg/mL. In addition, linear regression analysis also showed a positive correlation between BMI and plasma RBP4 levels (Spearman’s ρ = 0.24; *p* < 0.05).

Genetic profiling of *ABCA4* in this study confirmed that many known variants are present in the current AMD population and revealed 13 novel variants, which may be specific variants in this population. Seven of the thirteen variants were associated with an elevated RBP4 level in AMD participants, particularly in the participants with AREDS categories 3 and 4. Nevertheless, logistic regression analysis showed that the participants with the linked *ABCA4* variants had no significant association with severe AMD. Further investigation of the potential genetic contribution to elevated RBP4 levels in patients with severe AMD is warranted.

Our study analyzed RBP4 levels in the two different AMD groups and found a positive trend of higher plasma RBP4 levels in participants with severe forms of AMD. We also found that a plasma RBP4 level of 36 µg/mL or higher was associated with higher odds of severe AMD. However, due to difficulties in recruiting age-matched healthy participants, there was a significant age difference between the severe AMD and healthy/mild AMD groups. As a result, the strong correlation between age and AMD severity overshadowed the correlation between RBP4 levels and AMD severity in this exploratory investigation.

A recent study also compared the plasma RBP4 levels in healthy controls in various age groups and patients with geographic atrophy. The authors concluded that there was no significant association between RBP4 level and age or geographic atrophy. However, the study was limited by the enrolled participants (10 in each age group). Therefore, whether there is an association between RBP4 levels and AMD severity warrants further larger-scale investigations.

An RBP4 inhibitor, fenretinide, has been used in a clinical trial to treat non-neovascular AMD patients [35]. In this 2-year randomized controlled trial, 246 AMD patients with only geographic atrophy were treated with 100 mg or 300 mg of fenretinide or placebo. The results showed a reduction in the incidence of choroidal neovascularization onset in the treated patients, especially in the higher dose group. Moreover, in patients who achieved RBP4 levels below 1 μM (approximately 20 µg/mL) after fenretinide treatment, a lower serum RBP4 level was correlated with a slower rate of geographic atrophy progression. Although the overall cohort showed no statistically significant retardation of the growth rate of geographic atrophy due to an insufficient number of participants achieving a serum RBP4 level less than 1 μM, a further clinical trial targeting RBP4 is ongoing (clinicaltrials.gov identifier: NCT03735810).

Our study has several limitations, including a small number of patients, a lack of age matching, and a cross-sectional design. The lack of age matching between groups was largely due to the inherent limitation of the recruitment process. The recruitment of age-matched patients for all AMD severity grades in the future can eliminate the overwhelming effect of age on AMD severity and further elucidate the role of RBP4. A longitudinal follow-up of this cohort with qualitative measurement of AMD lesions, including drusen burden and geographic atrophy, can also provide a more in-depth understanding of this factor.

## 5. Conclusions

The current pilot study found that several linked *ABCA4* variants were associated with a significantly higher RBP4 level, and a higher RBCA4 level was related to severe AMD in univariate analyses. As age remains the strongest risk factor for severe AMD, and the severe AMD group in the current study was significantly older in age than the healthy/mild AMD group, the above relationship between RBP4 and AMD severity became nonsignificant after adjustments for age, sex, BMI, and metabolic syndrome. Nevertheless, the role of RBP4 and *ABCA4* variants in AMD pathogenesis and therapeutics should be further pursued in age-matched studies.

## Figures and Tables

**Table 1:** Demographics and clinical characteristics.

	Healthy/Mild AMD Group	Severe AMD Group	*p* Value
*n* =	32	35	
Female, No. (%)	22 (68.8)	22 (62.9)	0.80
Age, mean ± SD, y	58.8 ± 7.4	72.6 ± 7.7	<0.0001
BMI, mean ± SD, kg/m^2^	23.3 ± 3.5	23.8 ± 2.9	0.53
Obesity, No. (%)	1 (3.1)	0 (0)	0.48
Metabolic syndrome, No. (%)	9 (28.1)	18 (51.4)	0.08
Cardiovascular diseases, No. (%)	0 (0)	0 (0)	NA
Age at AMD diagnosis, mean ± SD, y	64.6 ± 7.7 ^1^	69.7 ± 7.5	0.06
AMD duration a, mean ± SD, y	1.1 ± 2.0 ^1^	2.9 ± 4.0	0.046
Plasma RBP4 level, mean ± SD, µg/mL	30.4 ± 7.0	36.8 ± 8.3	0.0012

AMD, age-related macular degeneration; BMI, body mass index; RBP4, retinol binding protein 4. ^1^ Healthy participants were excluded from this analysis.

**Table 2:** List of the identified *ABCA4* gene variants in participants with AMD.

dbSNP ID	Variant	Protein Change	Variant Type	Allele Frequency (%)			Severe AMD, No./Total (%)	Plasma RBP4 Level ^d^
				Heterozygosity ^a^	Present Study ^b^	Reported in the Database ^c^		
rs3747961	c.*372A>G	NA	3 Prime UTR variant	100%	4.3	7.12	2/2 (100)	31.4 ± 4.7
rs6666652 ^3,4,7^	c.6764G>T	p.S2255I	Missense	100%	8.5	15.96	4/4 (100)	34.8 ± 7.0
rs1800717 ^3^	c.6730-3T>C	NA	Intronic	100%	8.5	13.61	4/4 (100)	34.8 ± 7.0
rs763108716	c.6579C>T	p.F2193=	Synonymous	100%	2.1	0.01	1/1 (100)	34.9
rs185601596	c.6479+19G>A	NA	Intronic	100%	2.1	0.01	1/1 (100)	47.8
rs17110761 ^3^	c.6282+7G>A	NA	Intronic	100%	19.2	12.42	8/9 (89)	33.3 ± 9.7
rs61748519 ^4^	c.6255C>T	p.L2085=	Synonymous	100%	4.3	0.35	1/2 (50)	37.5 ± 14.5
rs1801359 ^4,6^	c.6249C>T	p.I2083=	Synonymous	100%	19.2	12.42	8/9 (89)	33.3 ± 9.7
rs145766145 ^5^	c.4253+13G>A	NA	Intronic	100%	6.4	0.17	1/3 (33)	34.6 ± 6.0
rs76258939	c.3626T>C	p.M1209T	Missense	100%	6.4	0.18	1/3 (33)	34.6 ± 6.0
rs200551567	c.2841C>T	p.D947=	Synonymous	100%	2.1	0.01 ^e^	1/1 (100)	28.4
rs754765164	g.63395A>G	NA	Intronic splice acceptor variant	100%	2.1	0.002 ^e^	1/1 (100)	40.3
rs201602424 ^4^	c.1614C>T	p.A538=	Synonymous	100%	2.1	0.04	1/1 (100)	38.8
rs564661476	c.1356+10_1356+11insA	NA	Intronic insertion	26%	4.3	NA ^f^	2/2 (100)	30.9 ± 6.6
rs4147831 ^4,6^	c.1269C>T	p.H423=	Synonymous	90%	21.3	9.05	9/10 (90)	31.7 ± 6.3
rs6657239 ^4^	c.635G>A	p.R212H	Missense	100%	4.3	5.27	2/2 (100)	31.1 ± 11.4
rs2297632	c.2653+23C>G	NA	Intronic	100%	4.3	0.24	2/2 (100)	34.0 ± 8.8
rs1801555 ^4,6^	c.6285T>C	p.D2095=	Synonymous	94%	34.0	25.97	13/16 (81)	35.8 ± 8.1 ^g^
rs1762114 ^4,6^	c.6069T>C	p.I2023=	Synonymous	39%	91.5	80.22	35/43 (81)	34.2 ± 8.1
rs4147863 ^h^	c.6006-16G>T	NA	Intronic	90%	21.3	16.68 ^e^	8/10 (80)	37.8 ± 7.7 ^g^
rs2275029 ^h,4,6^	c.5844A>G	p.P1948=	Synonymous	90%	21.3	19.79	8/10 (80)	37.8 ± 7.7 ^g^
rs1800739 ^h^	c.5836-11G>T	NA	Intronic	90%	21.3	20.36	8/10 (80)	37.8 ± 7.7 ^g^
rs4147857 ^h,4,6^	c.5814A>G	p.L1938=	Synonymous	90%	21.3	20.45	8/10 (80)	37.8 ± 7.7 ^g^
rs4147856 ^h^	c.5715-25A>C	NA	Intronic	90%	21.3	20.48	8/10 (80)	37.8 ± 7.7 ^g^
rs1801574 ^h,4,6^	c.5682G>C	p.L1894=	Synonymous	90%	21.3	23.75	8/10 (80)	37.8 ± 7.7 ^g^
rs55860151	c.4774-17_4774-16del	NA	Intronic	74%	66.0	17.0 ^e^	22/31 (71)	33.5 ± 7.7
rs1800549 ^1^	c.4283C>T	p.T1428M	Missense	100%	17.0	0.34	5/8 (63)	35.1 ± 10.3
rs3112831 ^4^	c.1268A>C	p.H423R	Missense	92%	51.1	26.04	20/24 (83)	35.8 ± 8.4
rs4147830	c.1240-14C>T	NA	Intronic	60%	85.1	47.22	29/40 (73)	32.9 ± 7.8
rs2297634 ^2^	c.302+26A>G	NA	Intronic	57%	89.4	49.54	30/42 (71)	33.6 ± 8.0
rs4847281 ^6^	c.141A>G	p.P47=	Synonymous	68%	76.6	98.91	28/36 (78)	33.5 ± 7.6

AMD, age-related macular degeneration; NA, not applicable; RBP4, retinol binding protein 4. ^a^ Percentage of carriers who carry a heterozygous genotype. ^b^ Forty-seven AMD participants in the present study. ^c^ Reported by gnomAD, unless otherwise specified. ^d^ The RBP4 level was calculated for all participants with the variant. Several variants were only found in a single participant; therefore, no SB was shown. ^e^ Reported by the TOPMed database. ^f^ No frequency reported by the gnomAD or TOPMed databases. ^g^ Significantly elevated RBP4 levels compared to participants without the variant. ^h^ Linked variants. ^1^ Allikmets et al., 1997 [11]. ^2^ Battu et al., 2015 [26]. ^3^ Fujinami et al., 2019 [27]. ^4^ Schulz et al., 2017 [28]. ^5^ Stenirri et al., 2004 [29]. ^6^ Zernant et al., 2011 [30]. ^7^ Valverde et al., 2006 [31].

**Table 3:** Logistic regression analysis of the factors associated with severe AMD.

	Unadjusted		Adjusted Model 1 ^a^		Adjusted Model 2 ^b^	
	OR (95% CI)	*p* Value	OR (95% CI)	*p* Value	OR (95% CI)	*p* Value
Age, y	1.26 (1.13–1.40)	<0.0001	1.27 (1.13–1.44)	<0.0001	1.27 (1.12–1.43)	<0.001
Female	0.77 (0.28–2.12)	0.612	2.03 (0.42–9.73)	0.377	2.17 (0.44–10.71)	0.340
BMI, kg/m^2^	1.05 (0.90–1.23)	0.524	0.99 (0.78–1.26)	0.938	1.00 (0.79–1.27)	0.999
Metabolic syndrome						
No	Reference		Reference		Reference	
Yes	2.71 (0.98–7.48)	0.055	0.52 (0.11–2.46)	0.411	0.60 (0.13–2.84)	0.514
Linked *ABCA4* variants ^c^						
No	Reference		-		-	
Yes	0.89 (0.29–2.73)	0.840	-		-	
Plasma RBP4 level, µg/mL	1.12 (1.04–1.20)	0.003	1.06 (0.96–1.17)	0.257	-	-
Plasma RBP4 cutoff levels						
32 µg/mL	3.18 (1.17–8.70)	0.0235	-	-	-	-
33 µg/mL	4.22 (1.52–11.76)	0.0058	-	-	-	-
34 µg/mL	5.75 (1.99–16.67)	0.0012	-	-	-	-
35 µg/mL	6.06 (2.04–17.86)	0.0011	-	-	-	-
36 µg/mL	8.13 (2.51–26.32)	0.0005	-	-	3.75 (0.77–18.29)	0.102

AMD, age-related macular degeneration; BMI, body mass index; CI, confidence interval; OR, odds ratio; RBP4, retinol binding protein 4. ^a^ Adjusted for age, sex, BMI, metabolic syndrome, and plasma RBP4 level (µg/mL). ^b^ Adjusted for age, sex, BMI, metabolic syndrome, and plasma RBP4 cutoff level of 36 µg/mL. ^c^ The six linked *ABCA4* variants found in this study: rs4147863, rs2275029, rs1800739, rs4147857, rs4147856, and rs1801574.

## Data Availability

All data generated or analyzed during this study are included in this article. Further inquiries can be directed to the corresponding author.
